# Supplementary material for: Evaluation of the Influence of Intervention Tools Used in Nutrition Education Programs: A Mixed Approach
Source: Nutrients. 2025 Jul 28;17(15):2460. doi: 10.3390/nu17152460 (PMC12348812; doi:10.3390/nu17152460)
Supplement: Supplementary file 1 [file nutrients-17-02460-s001.zip › nutrients-3702984-supplementary.pdf]

Table S1. Summary table of selected studies.

First author (year)	Objectives	Population	Intervention methods	Lesson duration / Program length	Outcome	Questionnaire	Results
Antón-Peset, A. (2021)	to explore the degree of knowledge, awareness and attitudes towards FW generated in the school canteen	Primary school (Nursery to year 6), in Valencia (Spain)	Questionnaires, posters and recordings. Individual, small group and class activities: "The unsupportive lunch", group reflection, "Water in the world"	45'/3 weeks	FW Collection and Measurement	FW knowledge assessment questionnaire + Questionnaire for parents to monitor FW at home	Change in waste on plates and improvement in knowledge of and awareness about FW
Antwi, J. (2020)	to evaluate the effect of a NE intervention on the NK, attitude, practices, and nutrition status of school-age children, teachers and home-based caregivers	School-age children (6–12 y.o), caregivers and teachers, in Ghana	Teacher's training, one-day training for home-based caregivers, group discussions, group works, hands-on activities, workbook assignments, singing sessions and nutrition competitions	2h/6 weeks	Dietary intakes (24-hour recall and food frequency methods). Anthropometric data. Nutrition KAP scores and nutrition status	Sociodemographic survey + nutrition knowledge questions	The NE intervention produced statistically significant improvements in NK scores particularly among lower primary school children in the IG as compared to the CG.
Asakura, K. (2021)	to evaluate the effect of the education program and the change in attitude/ behavior toward diet	N.2650, children (5th and 6th graders, 10–12 y.o), in Kanto (Japan)	Lecture at school, teaching materials for homework, quizzes and two handouts	45'/3 months	Dietary assessment: NK and attitudes/behaviors toward diet	2 questionnaires: one for dietary assessment and one to measure NK and attitudes/behaviors toward diet + a self-administered diet history questionnaire (BDHQ for adults and BDHQ15y for school children and adolescents based on the Standard Tables of Food Composition in Japan)	The proportion of children who discussed diet with their guardians increased. Guardian attitudes did not clearly change. NK after the nutrition education program was higher than before
Belando-Pedreño, N. (2023)	to design an intervention program in intra- and interpersonal competencies together with NE and corporality	School children (8-14 y.o), in Spain	2 educational workshops. Nutri plate/Harvard plate and chef frog (an infographic who explained the difference between fast and healthy food)	1h30'	Satisfaction of children and adolescents	Ad-hoc questionnaire to evaluate participants perception of the educational intervention	High satisfaction with the educational program "MotivACTION"

First author (year)	Objectives	Population	Intervention methods	Lesson duration / Program length	Outcome	Questionnaire	Results
Bibiloni, M. D. M. (2017)	to evaluate the effectiveness of a NE programme to improve diet quality and to reduce obesity prevalence	Children (3-7 y.o) and parents	Cartoons, group discussion, drawings, creation of graphics, crosswords	15' (children), 30' (adults)/3 years	BMI difference between IG and CG, adherence to MD	KIDMED	Improvement in adherence to MD; increase in consumption of fruit and vegetables
Buyco, N. G. (2022)	to investigate the effectiveness of an intervention intended for school children and their mothers/caregivers in terms of improving the nutrition behavior of the children	N.385, child-mother pairs. Schoolchildren (7-9 y.o) and their mothers/ caregivers, in Laguna (Philippines)	4-week cycle menu, nutrition classes, teachers training	25'/60h	Changes in nutrition attitude/behavior and in food intake	Modified CADET (Child and Diet Evaluation Tool). The CADET questionnaire was given as homework to the children for their mothers/ caregivers to fill out.	The IGs obtained higher mean attitude scores than the non-intervention group from baseline to endline. IGs showed a significant increase in mean scores on attitude towards vegetables.
Calvo Malvar, M. (2021)	to assess the effects of the Atlantic diet on anthropometric variables, metabolic profile, and nutritional habits	N.250, families (720 adults and children), in A Estrada (Spain)	3 educational sessions, cooking classes, written supporting material, recipe book, a daylong cooking course given by a chef, food baskets every three weeks, the food pyramid	1 year	Anthropometric variables, lipid profile. Changes in inflammation markers, glucose and insulin resistance levels, systolic (SBP) and diastolic (DBP) blood pressure.	Socio Demographic survey	Increase in the IG in the consumption of fresh fruits and pulses, and less consumption of sugar-sweetened beverages
D'Adamo, C. (2016)	to determine whether an experiential NE intervention focusing on spices and herbs improves diet quality and healthy eating attitudes among adolescents more than standard nutrition education alone	N.110, students (grades 9-12) in Baltimore, (Maryland)	Lectures, gift cards, MyPlate, cooking and meal preparation, creation of novel recipes, group activities, spice taboo, interactive educational games, a notebook	1h/6 weeks (10h total)	Dietary intakes and attitudes toward healthy eating	Three-day food records + healthy eating attitudes questionnaire	Modest but significant improvements in whole grains and protein foods intakes and attitudes toward eating vegetables, whole grains, lean protein, and low-fat dairy
D'Egidio, V. (2021)	to teach healthy nutrition and physical activity and to motivate children to incorporate active and healthy lifestyle choices into their everyday life	Primary school children (grades 2nd and 3rd, 6–8 y.o)	Oral presentation, 3 game sessions, board and card games. Nutrition games: Pappa, Zompa Verdura: card games; Oca egiziana: board game; L'Egiziamo,	5 months	Assessment of physical activity and nutritional behaviour	Two multiple-choice pre- and post-intervention questionnaires	Effectiveness in changing nutrition and physical activity behaviour. Significant differences for both nutritional and physical activity interventions in behavior scores

First author (year)	Objectives	Population	Intervention methods	Lesson duration / Program length	Outcome	Questionnaire	Results
			nutrition pyramid: movement game - Piramidiamo. Physical activity games: Muovi-AMOCI-AMICI: board game; L'Egiziamo physical activity pyramid, Staffetta di gruppo, Il cerchio, Gli animali del bosco: movement game				
Del Río, N. G. (2019)	to improve the long-term health of children	Primary school children (6–12 y.o)	Weekly group session on healthy habits, physical activity etc; active video gaming (TANGO:H); physical activity with Wii Fit Plus. Education session for parents using Classdojo	2h/3 months (36h total)	Body composition, emotion during the intervention, behaviour and personality, children gaming profile	Multidimensional questionnaire on child's behaviour and personality + player profile test + KIDMED + nutrition questionnaire di Delgado/Tercedor	Significant improvement in knowledge of healthy nutrition, better habits and satisfaction. Slight improvement in the KIDMED
Diamantis, D. (2023)	to present a best practice example of a lifestyle school-based training program, the Nutritional Adventures	N.12.451, primary school's students, in Greece	online educational intervention + at-home tasks, puzzles, crossword puzzles, quizzes, leaflets, and diaries, nutrition fairytale, interactive training sessions	1h/1 months	Dietary habits, physical activity status, children's HRQoL	Sociodemographic, dietary and other lifestyle habits questionnaire	Significant increase in MD adherence, improvement in KIDMED score
Elmas, C. (2020)	to assess the effect on students of NE	N.27, fourth- and fifth-grade teachers and 718 students, in Famagusta (Cipro)	in class, by teachers and researchers, book and powerpoint	40'/3 weeks	NK	Short general information questionnaire for all participants + NK questionnaire for teachers + pre and posttest healthy nutrition questionnaire for students	The results showed that there were significant differences between the pre- and posttest scores of both teachers and students
Eustachio Colombo, P. (2020)	to shape children's diet and reduce food related to GHG emissions	3 primary schools (grades 0-9), in Sweden	Introduction of an optimized school lunch menu for 4 weeks	1 week/1 month	Effects of FW, consumption and pupils' satisfaction with the meals	Online questionnaire assessing the lunch satisfaction both at baseline and during the intervention	Optimization resulted in a food list that was 40% lower in GHGE, met all nutrient recommendations for school meals, and cost 11% less
Fernández Álvarez, M. D.	to assess the feasibility of an educational intervention on	N.319, adolescents (14-19 y.o), in	Posters, food wheel, food pyramid, web app, group	N/A	Participants engagement	KIDMED + 5 questions' final self report survey	Improvement of health eating habits. Positive effect of the

First author (year)	Objectives	Population	Intervention methods	Lesson duration / Program length	Outcome	Questionnaire	Results
M. (2020)	adherence to healthy eating habits	Spain	activities, recipes preparation				intervention on the KIDMED score
Franciscato, J. S. (2019)	to evaluate the results of the “Nutriamigos®” Program in the knowledge about food and nutrition of children of different socioeconomic levels, gender, age and Body Mass Index	N.341, students (six- to ten-year age group), in São Paulo (Brazil)	DVD of five cartoon episodes of 11 minutes each, food pyramid, posters, games and paintings	50'/3 months	Weight, Height, Body Mass Index, nutrition and dietary knowledge	NK questionnaire, applied before and after intervention	Knowledge about diet and nutrition improved significantly
Gianfredi, V. (2024)	to raise awareness on healthy eating, through theatrical and practical workshops	Children (5-12 y.o)	Educational laboratories, cooking classes, theatrical workshops, interactive games, theatre laboratories, healthy snacks and gadgets	2 hours once a month for 4 months + once a week for 5 months/ 1 year	Children’s knowledge on nutrition and their food-related behaviour	KIDMED + ZOOM8 questionnaire	Statistically significant improvement in MD adherence among children and positive trends in some families’ food habits
Grosso, G. (2024)	to provide a multidimensional intervention in school children and adolescents to increase awareness, knowledge, and adherence to the MD	Children and adolescents (6-17 y.o) living in 5 Mediterranean countries (Spain, Portugal, Italy, Egypt, and Lebanon)	Formal education, didactic materials. Non formal education, newly designed global MD-based line of educational material, development of new snacks and recipes reformulation, web/mobile app development, physical activities	6 months	Changes in adherence to the MD, physical fitness and body composition.	Preliminary consumer electronic survey aiming to assess the general level of adherence to the Mediterranean Diet. KIDMED.	
Herawati, H. D. (2024)	to compare preschoolers’ fruit and vegetable preferences, fiber intake, and consumption of high-calorie food before and after a gardening-cooking intervention	Pre-school children (4-6 y.o)	Gardening activities, cooking activities	9 session / 3 months	Attitudes, fruit and vegetable preferences, and food intake	Attitude questionnaire	In the IG: fruit preference, vegetable preference, and fiber intake significantly increased after intervention
Jobet, E. (2024)	to evaluate adherence to MD	Pre-school age (≥18 m.o) and parents	Infographics, didactic material.	5 months	Paediatric and nutritional visits	Socio Demographic survey + KIDMED	Improved consumption of fruit, vegetables, pulses, fish and dried fruit; improvement in adherence to MD and good correlation between

First author (year)	Objectives	Population	Intervention methods	Lesson duration / Program length	Outcome	Questionnaire	Results
							children and parents' adherence
Jung, T. (2019)	to examine the effectiveness of a school-based healthy eating intervention program on the improvement of healthy eating knowledge and healthy food choice behavior among elementary school students	N.1472, school children (646 kindergartners to 2nd graders and 826 3rd to 5th graders)	Healthy food literacy lectures, physical education classes, cafeteria activities, games, traffic light theme, visual art, music, reading, charts creation	1 year	Improvement of health literacy in the choice of healthy food among elementary students	Two survey questionnaires developed for pre- and post assessments for two different age groups	Improvements in their food literacy, especially in their responses on French Fries, frozen yogurt, broccoli, bacon, banana, and green beans
Karpouzis, F. (2024)	to evaluate FEAST effect on children's fruit and vegetable (F&V) intakes	N.977, children (5th and 6th grade, c.a 11 y.o), in Australia	Face-to-face or online lectures by school teachers, designing recipes, collating a class cookbook, cooking meals, tasting foods prepared with classmates, families and/ or volunteers	90'/10 weeks	Evaluation of FEAST effect on children's fruit and vegetable (F&V) intakes, F&V variety consumed, NK, food preparation/cooking skills, self- efficacy and behaviours, food waste knowledge and behaviours, and food production knowledge	Custom designed survey with general students' data + 25-item self-reported survey to evaluate intervention implementation both in teachers and students	No evidence was found for improved F&V intakes in children, nor secondary outcomes
Kendel Jovanovic, G. (2023)	to investigate the effect of the educational program on the NK, diet quality, lifestyle, and nutritional status of school children	School children (10–12 y.o), in Croatia	Presentations, brochures, infographics, posters, website, school meetings	20'/ 3 weeks + 6-9 weeks follow up	Association between physical activity and other healthy lifestyle habits, including appropriate nutrition and MD adherence	Sociodemographic, habits, screen time use and NK questionnaire	Their NK significantly increased after the educational program, especially about daily servings of fruits and vegetables
Keshani, P. (2016)	to assess the effect of a school-based NE program on nutritional status of primary school students	N.221, primary school children (9.5-10.5 y.o), in Shiraz (Iran)	NE sessions for students and for mothers, lectures, problem solving, goal setting, games, entertainment and competition, animations, making story and targeted snacks, posters, writing story, draw painting, healthy snacks, pamphlets, booklets	2h/ 1 year	Anthropometric indices including weight, height and waist circumference	Socioeconomic questionnaire + NK questionnaire	The anthropometrics and NK of participants in both intervention and control group was significantly increased

First author (year)	Objectives	Population	Intervention methods	Lesson duration / Program length	Outcome	Questionnaire	Results
Kim, J. (2019)	to describe the modifiable dietary risk factors for nutritional management in childhood obesity	Children (7-16 y.o)	Face-to-face training, weekly nutrition sessions, monthly lifestyle education sessions, food preparation / cooking classes, sessions with parents, sessions with dietitians, fruit intervention	30'/6 months	Weight fluctuation, increased energy intake, macronutrient intake, unhealthy dietary behaviors	Food frequency questionnaire	Dietary intervention with a multisectoral approach has had positive outcomes in modifying obesity-related dietary risk factors for obese children and adolescents
Lee, K. A. (2016)	to measure the changes in attitudes towards, and the behavioral intention of making healthy food choices	Middle school students (6th grade), in New Jersey	School lectures, field trip to local community farm, practical activities, hands-on farm chores, poster boards, kitchen herb garden	80'	Social Cognitive Theory (SCT) variables (behavior intention, attitude, historical social norm, physical and social environment, knowledge, outcome expectations and self-efficacy	Pretest and posttest surveys	Positive behavior change
Li, S. R. (2024)	to develop a NE intervention to promote healthy eating evaluate the effectiveness of this intervention on healthy eating knowledge, attitude and behaviour	N.75, primary school students (3rd grade, 9-10 y.o), in Taiwan	Video, group discussion, homework assignments, worksheets, lectures	40'/1 month	'Healthy Eating Knowledge', 'Healthy Eating Attitudes', 'Healthy Eating Behaviour' questionnaires	Self-administered questionnaire to assess Healthy Eating Knowledge intervention effectiveness	The intervention had no significant effect on the mean overall score for healthy eating knowledge or behaviours
López-Gil, J. (2023)	to determine effects of a lifestyle-based intervention on adherence to 24-h movement behaviors and MD in schoolchildren and parents'/guardians ("halo" effect)	School children (6-13 y.o)	Infographics, video recipes, brief video clips, information pills	6 months	Changes in 24-h movement behaviors and adherence to the MD. PA and sedentary time and screen time	KIDMED + self-report questionnaire called the Youth Activity Profile—Spain (YAP-S), + self-report questionnaire of the PACO (Pedalea y Anda al Cole) project	
Maatoug, J. (2015)	to evaluate the effectiveness of a school-based behavioral intervention on overweight and obesity rates	Middle school students (11-16 y.o), in Sousse (Tunisia)	PowerPoint + Q&A sessions, organization of open days, posters, flyers, dances, skits, teachers training, interactive lessons, after-school soccer games	3 years	Reduction in overweight and obesity rates	Standardized questionnaire to evaluate physical activity behaviors and fruit and vegetable consumption	Significant increase in fruit and vegetable intake by the IG; decrease in overweight
Mahmood, M.	to explore the impact of	N.70, primary	Classes, video, workshops,	90'/12 weeks	Children's eating habit	KIDMED	The mean level of adherence to the

First author (year)	Objectives	Population	Intervention methods	Lesson duration / Program length	Outcome	Questionnaire	Results
A.	family-based NE intervention program on eating habit among obese children	school children (3-6 grades, 8-12 y.o)	games, physical activity		(level of adherence to MD)		MD increased, indicating the effectiveness of the intervention program
Martins, M. L. (2016)	to determine and compare the effect of two interventions in reducing the plate waste of school lunches	N.144, primary school children (fourth grade), in Porto (Portugal)	NE sessions in classrooms, oral presentations, planning of a school week menu, posters, 'No Plate Waste Day', debate session, flyers	6h	NE and food wast behaviour		Reduction of FW in the short term by children and in the medium term by teachers
Mogre, V. (2024)	to evaluate the effects of a school-based food and NE intervention on the nutrition-related knowledge, attitudes, anthropometric indices, dietary habits and physical activity levels of children	School-age children (10–12 y.o) in Tamale	Tripartite approach to NE involving the family, community and the school. Nutrition information, reading aloud, active discussions, nutrition games, charades, and artwork outdoor exercises, videos, healthy snacks, family newsletter, drawing, MyPlate, food traffic system, colourful posters, family newsletters	60'/6 weeks	Anthropometric measurements of weight and height, percentage body fat, waist circumference, nutrition related knowledge and attitudes, physical activity levels, dietary habits	Attitude towards nutrition questionnaire + physical activity questionnaire for children (PAQ-C). Consumption of vegetables was evaluated by 3 specific question	The intervention significantly improved the nutrition-related knowledge of children in the IG and the number of days they ate fruits in a week
Morelli, C. (2021)	to investigate the impact of nutrition education program (NEP) on the adherence to the MD (mediterranean diet) and on the inflammatory status in healthy adolescents	N.85, adolescents (14–17 y.o)	Seminars, interactive laboratories, official website, Facebook page	35'/1 year	Adherence to MD, intensity of PA levels, anthropometric measurements	Oral interview with nutritionists through a nutritional history record + KIDMED	Adherence to the MD evaluated by KIDMED score increased in all adolescents
Ng, C. M. (2024)	to evaluate the effectiveness of a culinary nutrition education program in terms of children's dietary practices and variety	N.83, children (10–11 y.o), in Kuala Lumpur (Malaysia)	Hands-on practical culinary sessions, storytelling, meal tasting, booklet, a healthy food ingredient, measuring cups, and spoons to encourage their involvement in meal preparation at home	1h/12 weeks	Children's dietary practices and dietary variety	Children's dietary practices adapted guided form in view of the Malaysian Dietary Guidelines for Children and Adolescents	Beneficial outcomes in children's increased frequency consumption of healthful foods, along with a reduction in less healthful foods, even 3 months after the intervention

First author (year)	Objectives	Population	Intervention methods	Lesson duration / Program length	Outcome	Questionnaire	Results
Ogunsile, S. E. (2016)	to improve knowledge, attitude and practice of healthy eating	N.143, adolescents (mean age of 13.59 ± 1.49 y.o), in Ibadan (Nigeria)	board game, games	80'/8 weeks		Questionnaire used to assess knowledge, attitude and practice of healthy eating (ADKHEQ) (ADAHEQ)(ADPHEQ)	In this study, adolescents exposed to board game NE had higher posttest mean knowledge, attitude and practice scores than those in the CG
Patra, E. (2023)	to explore and map the SHD indicators used to date in primary school interventions	Primary school students (5-12 y.o)	Nutritionist session, teacher trainings, school gardening, poster creation, local farm visits, parent trainings				Results showed that indicator definitions and measuring methods were not harmonized across research efforts
Raut, S. (2024)	to assess the effect of NE on NK, attitude, and diet quality among school-going adolescents	N.226, students (grades 6 to 10, 12-19 y.o), in Banepa (Nepal)	Mini-lectures, interactive discussions, textbooks, posters, leaflets, pamphlets, educational videos, PowerPoint	45'/12 weeks	Change in NK, nutrition attitude and diet quality	Face-to-face interviews with pre-tested semi-structured questionnaires + nutrition knowledge, attitude, and diet quality questionnaire	
Roccaldo, R. (2017)	to assess the effects of a NE program	N.494, primary school students (fourth grade), in Italy	In class lessons	1h/6 weeks	Adherence to MD and BMI before and after the intervention	KIDMED	Improvements in the adherence to the MD in the total sample. Significant increase in the proportion of children who improved their frequency of consumption of fruit and vegetables
Roset-Salla, M. (2016)	to evaluate the effectiveness of an educational programme on healthy alimentation, carried out in day-care centres and aimed at the parents of children from 1 to 2 years of age, regarding the acquisition of healthy eating habits among themselves and their children	Children (from 1 to 2 y.o) and their parents	4 theoretical/practical educational workshops, games "go to the market" and "menu of the week", card game	90'/6 months		KIDMED and an FFQ to calculate macro- and micronutrients	The results of the present study showed significant increases in the adherence of parents to the MD, with regard to children the changes were less evident, and only an improvement in adherence to the MD was observed

First author (year)	Objectives	Population	Intervention methods	Lesson duration / Program length	Outcome	Questionnaire	Results
Saaka, M. (2021)	to evaluate the apparent effect of an ongoing NE intervention on nutrition related knowledge, attitudes, and consumption of diversified diets	N.232, children (6 - 36 months) and their families, in northern Ghana	Flip chart materials, gardens, game or ice breaking activities, preparation meals, pictures, cooking demonstration	2h/7 months	Nutrition-related knowledge and consumption of fruits and vegetables, 24-hour dietary recall	Interviews	NE among families with home gardens resulted in a greater mean higher nutrition related knowledge and attitudes of mothers and fathers
Saha, S. (2023)	to assess the effectiveness of a behavior-and age-specific NE intervention to improve NK and preference for F&V among elementary school children	N.150, primary school children	(USDA) MyPlate, cooking demonstrations, class-based NE, cooking and tasting activities, group activities, discussion sessions, watching videos, recording serving of F&V intake, handouts and recipes, PowerPoint presentations, reading, stickers with F&V	45'/6 weeks	General knowledge, F&V health benefits and intakes	A structured questionnaire to assess the changes in nutrition knowledge and F&V preference in children	Participants showed significant improvements in general knowledge related to F&V, serving size, and health benefits of F&V intake after the NE intervention
Scherr, R. (2013)	to provide a framework for implementation of multicomponent, school-based nutrition interventions	N.490, elementary school students (fourth grade and their parents and teachers), in California	Lessons, workshop, cookteachersing demonstrations, recipes, family newsletter, school garden, health fair	1h/ (15 sessions)	Dietary and nutrition knowledge and behavior, critical thinking skills, healthy food preferences and consumption, physical activity, digital photography of plate waste, anthropometrics	NK questionnaire + a food frequency questionnaire + a vegetable preferences assessment tool + the Test of Basic Science Process Skills + School and Community Actions for Nutrition survey + Parenting Practices Questionnaire	
Szczepan ́ska, E. (2022)	to assess the change in knowledge of the principles of healthy eating among children who followed the "Colourful means healthy" project	N.317, children (7-9 y.o), in Katowice	Sensory laboratory and technology laboratories, workshops, cooking, food pyramid, crosswords and puzzles, an original educational game	2 years	NK	Self-administered questionnaire created for the evaluation	There was a statistically significant difference between the percentage of correct answers provided by the pupils before and after NE
Taniguchi, T. (2022)	to implement the Food Resource Equity and Sustainability for Health (FRESH) study, a culturally based farm-to-school	Children and their families	Farm-to-school nutrition and garden curriculum, online and in-person hybrid parent curriculum, farm-to-school menu modifications,	75'/6 months	Changes in dietary intake, body mass index (BMI), systolic blood pressure (adults only), health status, and food insecurity		Although the FRESH study did not improve BMI or other secondary outcomes among children, there were significant increases in vegetable intake.

First author (year)	Objectives	Population	Intervention methods	Lesson duration / Program length	Outcome	Questionnaire	Results
	intervention to increase vegetable intake among children and their families		reading, gardening, and indoor and outdoor sensory activities, family recipe kit, cooking activity, 12 short video, in-person night workshops				
Torre, G. (2016)	to increase knowledge on food pyramid, MD, consumption of fruits and vegetables and physical activity	Primary school children	card games and board games (memory, goose game, Piramidiamo), physical activity games		Food pyramid knowledge, MD adherence	Demographic information, knowledge of nutrition, feeding behaviour and physical activity questionnaire for children	
Xu, Y. (2022)	to evaluate the effects of school- based nutrition and health education on children's NK, dietary behaviors, dietary intake, and nutritional status	N.2066, primary school children (grades 2 to 6 (8-10 y.o)	NK courses, textbooks, training for teachers, face-to- face training sessions, online training sessions, class competition, painting and speech competitions, the Healthy Life Weekly Notes, physical activity, plating vegetables	40'/2 years	NK, dietary intake and dietary behaviours	Student questionnaire based on the China National Nutrition and Health Surveillance + NK questionnaire + dietary intake questionnaire + dietary behaviour questionnaire	The results of this study suggest that school-based nutrition and health education may have a positive effect on NK, the frequency of eating breakfast, dietary intakes (not on nutritional status)
